# Prevalence of vitamin D deficiency and related factors among adults in Tokat province, Türkiye: a community-based cross-sectional study

**DOI:** 10.3389/abp.2025.15459

**Published:** 2026-01-07

**Authors:** Muzaffer Katar, Rıza Çıtıl, Yalçın Önder, Osman Demir

**Affiliations:** 1 Department of Medical Biochemistry, Gaziosmanpasa University Faculty of Medicine, Tokat, Türkiye; 2 Department of Public Health, Tokat Gaziosmanpaşa University Faculty of Medicine, Tokat, Türkiye; 3 Department of Biostatistics, Tokat Gaziosmanpaşa University Faculty of Medicine, Tokat, Türkiye

**Keywords:** vitamin D deficiency, prevalence, obesity, risk factors, public health

## Abstract

**Background:**

Vitamin D deficiency is a major public health concern, worldwide yet data on the adult population in Türkiye remain limited. This study aimed to determine the prevalence of vitamin D deficiency and its associated risk factors in Tokat Province, Türkiye.

**Methods:**

A population based cross-sectional study was conducted among 2,225 adults aged ≥20 years through multistage stratified cluster sampling from both urban and rural areas. Data were obtained via structured questionnaires, anthropometric measurements, and fasting blood samples. Serum 25-hydroxyvitamin D3 [25(OH)D3] levels were measured and categorized as deficient (<10 ng/mL), insufficient (10–20 ng/mL), or sufficient (>20 ng/mL). Multivariate logistic regression was used to identify predictors of deficiency.

**Results:**

The mean age was 47.2 ± 15.2 years, and 54.8% were women. Vitamin D deficiency was present in 38.7% and insufficiency in 46.4% of participants. Deficiency was significantly more common among women, the older adult, obese individuals, and those with lower education levels and chronic diseases (p < 0.05). In the adjusted model female sex (aOR 0.19, 95% CI 0.14–0.27), obesity (aOR 1.76, 95% CI 1.36–2.28), and lower education were independent predictors of deficiency.

**Conclusion:**

Vitamin D deficiency and insufficiency are highly prevalent among adults in Tokat Province, despite adequate sunlight exposure in the region. Public health strategies/interventions should focus on high-risk groups, prioritize particularly women, older adults, and obese individuals through screening, education, and targeted supplementation.

## Introduction

Vitamin D deficiency is recognized as one of the most common public health problems worldwide. Low levels of vitamin D have been associated with a wide range of chronic and infectious conditions, including, diabetes (Hyppönen et al., 2001), cardiovascular disease (Lee et al., 2008), cancer (Holick, 2006a), multiple sclerosis (VanAmerongen et al., 2004), osteoporoz (Lips, 2001), autoimmune diseases (Cantorna et al., 2004), and microbial infections (White, 2008).

The role of vitamin D was first noted by Whistler in the 17th century in relation to rickets (O’riordan and Bijvoet, 2014). By the early 1900s, its fat-soluble nature, its production through ultraviolet (UV) sunlight exposure, and its antirachitic properties were described. The discovery of the specific forms vitamin D2 (ergocalciferol) and provitamin D3 (cholecalciferol) followed in the 1930s (DeLuca, 2014).

Vitamin D plays a critical role in bone metabolism as well as in various cellular and immunological processes. Inadequate sun exposure due to cultural clothing styles or excessive use of sunscreens, along with insufficient dietary intake or lack of supplementation, are common reasons for this vitamin D deficiency (Lips, 2007). Tropospheric ozone has also been linked to reduced synthesis of vitamin D. Other factors such as skin pigmentation, age, body weight, malabsorption disorders, and conditions affecting vitamin D metabolism can also influence serum levels (Holick, 2006b).

Currently, plasma (or serum) 25-hydroxyvitamin D [25(OH)D] is considered the best indicator of vitamin D status (Manicourt and Devogelaer, 2008), reflecting total input from cutaneous synthesis, diet, and supplements (Holick, 2007). The circulating concentration of 25(OH)D represents the cumulative effect of dietary intake and sunlight exposure, and it is widely accepted as a reliable marker of vitamin D status (Holick, 1996).

Although there is no universal consensus on the optimal serum level of 25(OH)D, most experts define deficiency as a level below 20 ng/mL (Holick, 2007). It is estimated that vitamin D deficiency affects approximately 30%–50% of the general population worldwide, yet this widespread condition often remains undiagnosed and untreated (Lee et al., 2008). To date, there is limited data available regarding the vitamin D status of adults in Türkiye (Atli et al., 2005; Alagöl et al., 2000; Guzel et al., 2001; Erkal et al., 2006).

This study aimed to determine the prevalence of vitamin D deficiency and insufficiency among adults in Tokat Province, Türkiye, and to identify sociodemographic and lifestyle factors associated with low vitamin D status.

## Materials and methods

### Study design, population, and sampling

This population-based cross-sectional study was conducted in Tokat Province, Turkey, including both urban and rural areas. The target population comprised adults aged ≥20 years, which based on the report by the Turkish Institute of Statistics as of 31 December 2021, the population in the province is 602,567.

Sample size was calculated assuming a 35% expected prevalence of vitamin D deficiency, 3% margin of error, 95% confidence level, and a design effect of 2, yielding a minimum required sample of 1,955 participants. Using multistage stratified proportional cluster sampling, 85 clusters (52 urban, 33 rural) representing 50% of all Family Health Units (FHUs) were selected proportionally by age and sex according to the local population pyramid. Ultimately, data were collected from 2,225 participants.

**FIGURE 1:**
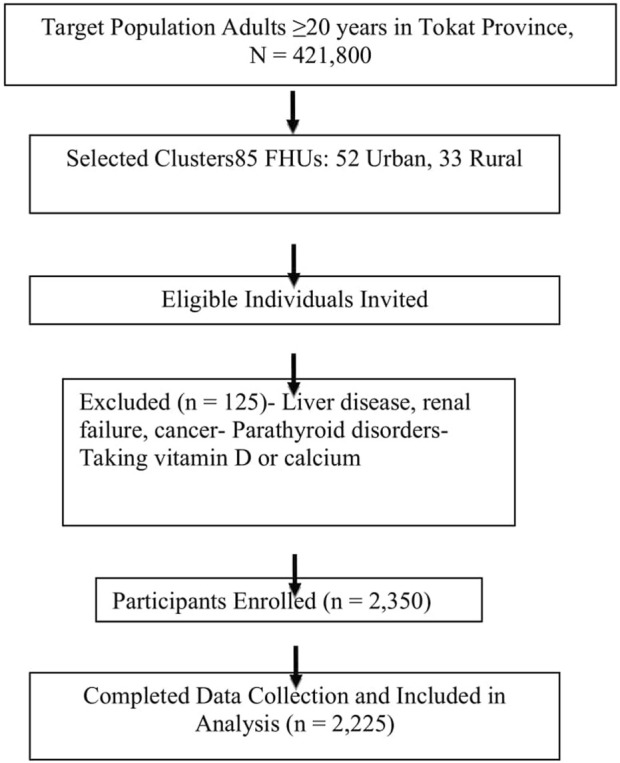
Flow diagram of participant selection, recruitment, and final data analysis.

Individuals with a history of liver disease, renal failure, cancer, parathyroid disorders, or who were taking calcium/vitamin D supplements were excluded from the study. More clearly shown in Figure 1.

### Data collection procedures

Data were collected between 10 April and 1 June 2013. However, given the time elapsed since data collection, changes in lifestyle, economy, and health awareness may have influenced current vitamin D status, which should be considered when interpreting these findings. Trained staff measured height, weight, waist and hip circumference using standardized protocols. Blood pressure was measured twice after a 15-min rest with a validated sphygmomanometer. Fasting blood samples were collected in the morning after an overnight fast of (at least 8 hours). All samples were stored at −80 °C. until analysis.

Participants completed a structured face-to-face interview that included sociodemographic characteristics, medical history, lifestyle factors, and dietary calcium intake.

### Biochemical analyses

Serum 25-hydroxyvitamin D3 [25(OH)D3] concentrations were determined using Roche Elecsys Vitamin D Total III assay kits on a Roche Cobas e 601 analyzer (Roche Diagnostics, Germany).

### Definitions

- Vitamin D Status: Categorized according to the 2020 Turkish Endocrinology and Metabolism Society (TEMD) guidelines as deficiency (<10 ng/mL), insufficiency (10–20 ng/mL), sufficient for bone health (>20 ng/mL), sufficient for extra-skeletal benefits (30–50 ng/mL), and intoxication (>150 ng/mL) (1 ng/mL = 2.5 nmol/L) (Prevention et al., 2003).
- Sunlight Exposure: Although direct measurement was not performed, data collection was conducted during spring, when average daily sunlight duration ranges from 6.2 to 8.0 h with temperatures between 6.7 °C and 27.0 °C. Most rural participants engaged in outdoor farming activities between 10 AM and 4 PM, generally without sunscreen.

### Statistical analysis

Continuous variables are reported as mean ± standard deviation, and categorical variables as frequencies and percentages. Group comparisons were performed using independent-sample t-tests or one-way ANOVA for continuous variables and chi-square tests for categorical variables. Multivariate logistic regression was used to identify factors associated with vitamin D deficiency. A two-tailed p-value <0.05 was considered statistically significant. Analyses were conducted using IBM SPSS Statistics version 20 (IBM Corp., Armonk, NY, United States).

### Ethics approval and permissions

The study protocol was reviewed and approved by the Clinical Research Ethics Committee of Tokat Gaziosmanpaşa University Faculty of Medicine (Approval No: 13-KAEK-024). Administrative permissions were obtained from relevant health authorities prior to data collection. The present study, which utilized these samples for vitamin D status analyses, approved by the Clinical Research Ethics Committee of Tokat Gaziosmanpaşa University Faculty of Medicine (Approval No: 21-KAEK-042). Researchers fully adhered to ethical guidelines throughout the study process, The study was supported by the Scientific Research Projects Unit (BAP) of Tokat Gaziosmanpaşa University for assay kits (Code number: 200/112).

## Results

A total of 2,225 adults participated in the study, including 1,220 (54.8%) women and 1,005 (45.2%) men. The mean age was 47.17 ± 15.23 years. Based on serum 25(OH)D3 levels, 38.7% of participants were classified as vitamin D deficient (<10 ng/mL), 46.4% as insufficient (10−20 ng/mL), and only 14.9% had sufficient levels (>20 ng/mL) (Table 1). These findings indicate that the majority of the population has suboptimal vitamin D status.

**TABLE 1:** Distribution of qualitative variables.

Variables	VitD status				p
	Total	Deficiency <10 ng/mL	Insufficiency 10–20 ng/mL	Sufficiency >20 ng/mL	
Vitamin D *(Main Exposure)*					
Serum vitamin D level; n (%)	2,225 (100)	861 (38,7)	1,033 (46,4)	331 (14,9)	​
Clinical parameters					
Height (cm)	162.31 ± 9.54	161.52 ± 9.59	162.79 ± 9.52	162.82 ± 9.33	0.009
Weight (kg)	75.95 ± 14.82	75.39 ± 14.97	76.17 ± 14.72	76.72 ± 14.73	0.309
Waist (cm)	106.25 ± 11.29	106.59 ± 11.56	106 ± 11.08	106.14 ± 11.24	0.521
WHR	0.89 ± 0.08	0.88 ± 0.08	0.89 ± 0.09	0.89 ± 0.08	0.213
BMI (kg/m2)	28.88 ± 5.53	28.96 ± 5.77	28.78 ± 5.39	28.98 ± 5.34	0.731
sBP (mmHg)	12.41 ± 2.25	12.48 ± 2.22	12.36 ± 2.26	12.37 ± 2.33	0.504
dBP (mmHg)	7.74 ± 1.27	7.81 ± 1.24	7.7 ± 1.26	7.69 ± 1.36	0.113
Obesity					
Normal	1,367 (61.4)	450 (52.3)a	678 (65.6)b	239 (72.2)b	<0.001
Obese (BKİ >= 30)	858 (38.6)	411 (47.7)a	355 (34.4)b	92 (27.8)b	​
Chronic disease					
Absent	1,054 (47.4)	347 (40.3)a	524 (50.7)b	183 (55.3)b	<0.001
Present	1,171 (52.6)	514 (59.7)a	509 (49.3)b	148 (44.7)b	​
Sociodemographic					
Age (year) ± SD	47.17 ± 15.23	48.4 ± 15.95	46.44 ± 14.81	46.29 ± 14.47	0.011
Gender; n (%)					
Woman	1,220 (54.8)	737 (85.6)a	406 (39.3)b	77 (23.3)c	<0.001
Man	1,005 (45.2)	124 (14.4)a	627 (60.7)b	254 (76.7)c	​
Age group (year); n (%)					
20–39	889 (40)	342 (39.7)	408 (39.5)	139 (42)	<0.001
40–64	988 (44.4)	348 (40.4)a	494 (47.8)b	146 (44.1)ab	​
65+	348 (15.6)	171 (19.9)a	131 (12.7)b	46 (13.9)ab	​
Geographic localization; n (%)					
Urban	1,299 (58.4)	524 (60.9)	583 (56.4)	192 (58)	0.149
Rural	926 (41.6)	337 (39.1)	450 (43.6)	139 (42)	​
Income status; n (%)					
<500 $	1,135 (51)	454 (52.7)	513 (49.7)	168 (50.8)	0.263
500–1,000 $	693 (31.1)	268 (31.1)	316 (30.6)	109 (32.9)	​
>1,000 $	397 (17.8)	139 (16.1)	204 (19.7)	54 (16.3)	​
Education					
Illiterate or literate. But no formal education	393 (17.7)	222 (25.8)a	139 (13.5)b	32 (9.7)b	<0.001
Formal education under high school	1,299 (58.4)	490 (56.9)	602 (58.3)	207 (62.5)	​
High school or above	533 (24)	149 (17.3)a	292 (28.3)b	92 (27.8)b	​
Marital status					
Married	1928 (86.7)	728 (84.6)	912 (88.3)	288 (87)	<0.001
Single	140 (6.3)	42 (4.9)a	67 (6.5)ab	31 (9.4)b	​
Widowed	157 (7.1)	91 (10.6)	54 (5.2)	12 (3.6)	​
Smoke					
Absent	1,383 (62.2)	677 (78.6)a	554 (53.6)b	152 (45.9)c	<0.001
Present	842 (37.8)	184 (21.4)a	479 (46.4)b	179 (54.1)c	​
Alcohol					
Absent	2017 (90.7)	834 (96.9)a	905 (87.6)b	278 (84)c	<0.001
Present	208 (9.3)	27 (3.1)a	128 (12.4)b	53 (16)c	​

Data are shown as mean ± standard devaition or frequency, percentage. Independent samples test or pearson chi-square test was used. (ab): In same row, Common letters indicate statistical insignificance.

Women were significantly more likely to be vitamin D deficient compared to men (p < 0.001). The prevalence of deficiency (<10 ng/mL) was highest among older participants: individuals with lower income and education levels tended to have lower vitamin D levels (p < 0.001). On the other hand widowed individuals had higher mean vitamin D levels than married or single individuals (p < 0.001) (Table 1).

Lifestyle factors negatively affect vitamin D levels. Participants who did not smoke or consume alcohol had lower vitamin D levels than smokers and drinkers (p < 0.001), On the other hand, there were no significant differences in mean height, weight, waist circumference, waist-to- hip ratio, or blood pressure among vitamin D groups (p > 0.05), Participants with obesity (BMI ≥30) had a higher prevalence of deficiency compared to normal-weight individuals (p < 0.001). Similarly, those with chronic diseases had significantly lower vitamin D levels than participants without chronic conditions (p < 0.001). Interestingly, although obesity is generally associated with lower vitamin D, some data suggested slightly higher levels among certain obese subgroups, highlighting possible confounding effects of age and lifestyle (Table 1).

Significant sex-based differences were observed in demographic and clinical variables. Female participants had higher rates of obesity and chronic diseases (e.g., hypertension, DM), whereas males showed higher smoking and alcohol use (p < 0.05) (Table 2).

**TABLE 2:** General features of the studied population by Gender.

Değişkenler	​	Gender		p
	Total	Female	Male	
Age (year)	47.17 ± 15.23	46.64 ± 15.19	47.83 ± 15.28	0.067
Height (cm)	162.31 ± 9.54	162.08 ± 9.49	162.58 ± 9.58	0.222
Weight (kg)	75.95 ± 14.82	75.77 ± 14.92	76.17 ± 14.71	0.524
Waist (cm)	106.25 ± 11.29	106.22 ± 11.21	106.29 ± 11.38	0.869
WHR	0.89 ± 0.08	0.89 ± 0.08	0.89 ± 0.08	0.773
BMI (kg/m2)	28.88 ± 5.53	28.89 ± 5.62	28.86 ± 5.42	0.898
sBP (mmHg)	12.41 ± 2.25	12.47 ± 2.28	12.34 ± 2.22	0.195
dBP (mmHg)	7.74 ± 1.27	7.76 ± 1.3	7.71 ± 1.24	0.328
Age group (year)				
20–39	889 (40)	506 (41.5)	383 (38.1)	0.223
40–64	988 (44.4)	523 (42.9)	465 (46.3)	​
65+	348 (15.6)	191 (15.7)	157 (15.6)	​
Geographic localization				
Urban	1,299 (58.4)	703 (57.6)	596 (59.3)	0.424
Rural	926 (41.6)	517 (42.4)	409 (40.7)	​
Income status				
<500 $	1,135 (51)	677 (55.5)a	458 (45.6)b	<0.001
500–1,000 $	693 (31.1)	365 (29.9)	328 (32.6)	​
>1,000 $	397 (17.8)	178 (14.6)a	219 (21.8)b	​
Smoke				
Absent	1,383 (62.2)	1,048 (85.9)a	335 (33.3)b	<0.001
Present	842 (37.8)	172 (14.1)a	670 (66.7)b	​
Alcohol				
Absent	2017 (90.7)	1,195 (98)a	822 (81.8)b	<0.001
Present	208 (9.3)	25 (2)a	183 (18.2)b	​
Education				
Illiterate or literate. But no formal education	393 (17.7)	330 (27)a	63 (6.3)b	<0.001
Formal education under high school	1,299 (58.4)	708 (58)	591 (58.8)	​
High school or above	533 (24)	182 (14.9)a	351 (34.9)b	​
Marital status				
Married	1928 (86.7)	1,040 (85.2)a	888 (88.4)b	<0.001
Single	140 (6.3)	54 (4.4)a	86 (8.6)b	​
Widowed	157 (7.1)	126 (10.3)a	31 (3.1)b	​
Obesity				
NORMAL	1,367 (61.4)	635 (52)a	732 (72.8)b	<0.001
Obese (BKİ >= 30)	858 (38.6)	585 (48)a	273 (27.2)b	​
Chronic disease				
Absent	1,054 (47.4)	505 (41.4)a	549 (54.6)b	<0.001
Present	1,171 (52.6)	715 (58.6)a	456 (45.4)b	​

Data are shown as mean ± standard devaition or frequency, percentage. Independent samples test or pearson chi-square test was used. (ab): In same row, Common letters indicate statistical insignificance.

### Multivariate analysis

In multivariate analysis, the factors that remained associated with vitamin D deficiency after adjustment were: Gender: Women had significantly higher odds of vitamin D deficiency compared to men (AOR 0.191; 95% CI 0.137–0.266; p < 0.001). Other factors such as age, smoking, alcohol, education, obesity, and chronic disease were not significant after being controlled simultaneously (p > 0.05).

The multinomial model shows: Women have a much higher risk of vitamin D deficiency, either in the <10 ng/mL category (AOR 20.38; 95% CI 13.78–30.13) and 10–20 ng/mL (AOR 2.27; 95% CI 1.61–3.20) than men (p < 0.001). Older age significantly increases the risk of severe deficiency (<10 ng/mL) (AOR 1.017 per year; 95% CI 1.006–1.029; p = 0.003). Low education was associated with a higher risk for <10 ng/mL (AOR 0.681; 95% CI 0.481–0.966; p = 0.031).

Obesity, chronic disease, smoking, and alcohol consumption were not significantly associated after adjustment (Tables 3, 4).

**TABLE 3:** Multivariate binary logistic regression analysis results for Vit D deficiency.

Variable	Univariate				Multivariate			
	p	Odds Ratio	95% confidence interval		p	Adjusted odds ratio	95% confidence interval	
			Lower	Upper			Lower	Upper
Age	0.252	1,005	0.997	1,012	0.079	1,009	0.999	1,019
Gender	<0.001	0.199	0.152	0.261	**<0,001**	0.191	0.137	0.266
Smoking	<0.001	0.457	0.361	0.579	0.820	1,033	0.781	1,366
Alcohol	<0.001	0.468	0.334	0.655	0.440	0.868	0.605	1,244
Education	0.006	0.718	0.565	0.911	0.153	-	-	-
Formal education under high school	<0.001	0.468	0.316	0.691	0.664	0.906	0.580	1,415
High school or above	<0.001	0.425	0.278	0.650	0.479	1,209	0.715	2,043
Marital status	0.176	1,173	0.931	1,477	0.829	1,029	0.797	1,327
Obesity	<0.001	1,764	1,364	2,282	0.124	1,241	0.943	1,633
Chronic disease	0.002	1,452	1,148	1,837	0.411	1,117	0.858	1,454

Reference categories: Gender: Male; Smoking: Yes; Alcohol: Yes; Education: Secondary school and above; Marital status: Married; Obesity: Obese; Chronic disease: Yes. Bold values indicate statistically significant associations (p < 0.05).

**TABLE 4:** Multivariate multicategory logistic regression analysis results for Vit D deficiency.

Variable		Univariate				Multivariate			
		p	Odds Ratio	95% confidence interval		p	Adjusted odds ratio	95% confidence interval	
				Lower	Upper			Lower	Upper
<10 ng/mL	Age	0.033	1,009	1,001	1,018	**0,003**	1,017	1,006	1,029
10–20 ng/mL	Age	0.876	1,001	0.992	1,009	0.394	1,004	0.994	1,015
<10 ng/mL	Gender	<0.001	19,606	14,264	26,949	**<0,001**	20,380	13,783	30,133
10–20 ng/mL	Gender	<0.001	2,136	1,608	2,837	**<0,001**	2,271	1,612	3,200
<10 ng/mL	Smoking	<0.001	4,333	3,305	5,680	0.761	0.948	0.671	1,339
10–20 ng/mL	Smoking	0.015	1,362	1,062	1,746	0.838	0.971	0.729	1,293
<10 ng/mL	Alcohol	<0.001	5,889	3,633	9,544	0.055	1,708	0.989	2,951
10–20 ng/mL	Alcohol	0.092	1,348	0.953	1,907	0.700	1,075	0.745	1,550
<10 ng/mL	Education	<0.001	2,135	1,633	2,791	**0,031**	0.681	0.481	0.966
10–20 ng/mL	Education	0.887	1,018	0.792	1,310	0.059	0.752	0.559	1,011
<10 ng/mL	Marital status	0.285	0.817	0.565	1,183	0.616	0.896	0.583	1,376
10–20 ng/mL	Marital status	0.534	1,125	0.776	1,633	0.519	1,136	0.771	1,674
<10 ng/mL	Obesity	<0.001	0.421	0.320	0.555	0.061	0.742	0.544	1,013
10–20 ng/mL	Obesity	0.027	0.735	0.560	0.966	0.255	0.849	0.640	1,126
<10 ng/mL	Chronic disease	<0.001	0.546	0.423	0.705	0.259	0.839	0.618	1,138
10–20 ng/mL	Chronic disease	0.149	0.833	0.649	1,068	0.544	0.920	0.701	1,206

Reference categories: Gender: Male; Smoking: Yes; Alcohol: Yes; Education: Secondary school and above; Marital status: Married; Obesity: Obese; Chronic disease: Yes. Bold values indicate statistically significant associations (p < 0.05)

Overall taken together, these results indicate that vitamin D deficiency is common across all age groups but is particularly prevalent among women, the older adult, individuals with lower socioeconomic status, those with chronic diseases, and obese individuals. The observed inverse relationship between age, income, education, and vitamin D levels underlines the potential impact of social determinants on vitamin D status in this population.

## Discussion

This study comprehensively assessed the relationship between serum vitamin D levels and a broad range of sociodemographic, behavioral, and clinical factors in a large adult population in Türkiye. Our findings demonstrate that vitamin D deficiency is highly prevalent and confirm that specific subgroups—including women, older adults, and individuals with obesity—are at significantly higher risk (Tables 1, 2, 4).

More than 85% of participants had serum vitamin D levels below 20 ng/mL, consistent with global evidence indicating widespread hypovitaminosis D even in countries with abundant sunlight exposure (Qureshi et al., 2024).

Gender emerged as the strongest independent determinant: women were significantly more likely to have both severe (<10 ng/mL) and moderate (10–20 ng/mL) deficiency of vitamin D than men, possibly due to cultural clothing practices, less outdoor activity, and hormonal influences (Kader et al., 2020). Sex-based differences in lifestyle and clinical characteristics may contribute to the higher prevalence of vitamin D deficiency observed among women. This finding aligns with prior studies demonstrating higher deficiency rates in females attributable to cultural clothing patterns, lower sun exposure, and metabolic differences (Choi et al., 2019). For example: These findings align with earlier studies reporting high prevalence of deficiency across Turkey. Founded a deficiency rate of nearly 75% in adults from the Aegean region, with women at higher risk due to clothing limiting sun exposure (Hekimsoy et al., 2010)

Occupational studies by Aykal et al. (2016) and Skarphedinsdottir et al. (2014) emphasizing that indoor working conditions revealed high deficiency rates among operating room staff and anesthesia care providers in Turkey, Iceland, and the US (Du et al., 2023; Chu et al., 2021; Zhao et al., 2017). Add literature related to gender hormones.

Age showed a nuanced pattern; although mean age did not differ significantly in crude comparisons, regression analysis revealed that younger and middle-aged adults had significantly lower odds of severe deficiency than older adults, in line with literature indicating reduced cutaneous synthesis and increased chronic disease burden in older adult populations (Gallagher, 2013). Obesity was also a significant factor, with obese participants having up to 76% higher odds of deficiency—likely due to vitamin D sequestration in adipose tissue (Pereira-Santos et al., 2015) —although this association weakened but remained significant after adjustment.

Behavioral factors such as smoking and alcohol use initially appeared inversely related to deficiency but lost significance after adjustment, reflecting confounding by lifestyle and socioeconomic patterns. Likewise, lower education level was associated with greater risk in crude analyses but did not remain significant in adjusted models. Chronic diseases were related to deficiency in univariate analysis but not independently, likely due to collinearity with age and obesity.

The health consequences of vitamin D deficiency extend beyond bone metabolism. It may play a role in neurodegenerative disorders: Knekt et al. (2010) demonstrated a significant inverse association between baseline vitamin D levels and Parkinson’s disease risk in a Finnish cohort over nearly three decades. Sato et al. (1997) and Evatt et al. (2008) likewise reported lower vitamin D levels and higher prevalence of deficiency in Parkinson’s patients compared to controls. In our research, it was found that the patient subgroup, who experienced chronic disease, had a lower rates of deficiency, possibly due to smaller sample size and greater sunlight exposure in our region, but the trend remains consistent with international evidence suggesting neuroprotective effects via antioxidative mechanisms and neuronal calcium modulation. Further research with larger cohorts and cerebrospinal fluid analyses is needed to clarify this potential link.

Multiple international studies support these findings. Hovsepian et al. (2011) in Iran reported a median 25(OH)D level of 18–21 ng/mL, with deficiency prevalence exceeding 50%, higher among women and younger adults, and worse during autumn–winter. Brot et al. (2001) in Denmark showed that vitamin supplementation and intentional sun exposure raised serum levels significantly, while Heidari and Mirghassemi (2012) found a strikingly high 70% deficiency rate in Iranian adults, again worse for women. Occupational studies by Aykal et al. (2016) and Skarphedinsdottir et al. (2014) revealed high deficiency rates among operating room staff and anesthesia care providers in Türkiye, Iceland, and the US, emphasizing that indoor working conditions and lifestyle factors are key contributors.

Childhood and adolescent data echo this pattern. Aypak et al. (2013) showed nearly universal deficiency in Turkish children, with stronger associations in females and pubertal adolescents, and identified potential links to insulin resistance. Ethnic disparities have also been reported: Ford et al. (2006) highlighted higher deficiency rates among Asian and Black Afro-Caribbean groups in the UK, especially among women. Sahota et al. (2004) showed moderate seasonal variation and a clear inverse correlation between 25(OH)D and PTH levels, underlining potential effects on calcium homeostasis.

Despite abundant sunlight in Türkiye, factors such as latitude (around 39°N), cultural dress codes, modern indoor lifestyles, and limited supplementation contribute to persistent deficiency. Moreover, there is no universal consensus on optimal serum 25(OH)D thresholds; the WHO defines levels below 10 ng/mL as deficient, while other organizations consider <20 ng/mL insufficient and <10 ng/mL as severe deficiency (Prevention, 2003).

Key strengths of our study include its large representative sample and robust multivariate analysis, adding valuable national data to the global literature. However, limitations include its cross-sectional design, absence of seasonal follow-up, and lack of detailed dietary assessments. Additionally, since data collection occurred only in spring and early summer, the prevalence of vitamin D deficiency may have been underestimated due to seasonal variation. We measured vitamin D levels during summer when they are expected to peak; future research should include winter–spring sampling and more precise tracking of dietary intake, supplementation, and sun exposure habits. In addition, future studies should collect more detailed data on sunlight exposure duration, use of protective clothing, and dietary sources of vitamin D to improve exposure assessment accuracy.

In conclusion, our findings reinforce that vitamin D deficiency is highly prevalent in Türkiye and globally, with higher risk among women, older adults, obese individuals, indoor workers, and populations with cultural or lifestyle-related sun avoidance. Preventive public health strategies should prioritize awareness, routine screening, safe sun exposure, and appropriate supplementation to address this modifiable but significant health burden.

## Conclusion

In summary, this large, population-based study confirms that vitamin D deficiency is highly prevalent in the adult population of Tokat, Türkiye, reflecting a broader national and global public health challenge. Our results highlight that women, older adults, and individuals with obesity are particularly vulnerable to severe deficiency, despite living in a region with sufficient sunlight.

Given the growing evidence linking vitamin D status not only to bone and musculoskeletal health but also to neurological, metabolic, and chronic diseases, effective public health measures are urgently needed. Regular screening, education on safe sun exposure, dietary improvements, and targeted supplementation strategies should be prioritized for high-risk groups.

Future research should be prioritized for high-risk groups. Public health authorities should also consider national vitamin D fortification policies, population-level screening programs, and awareness campaigns to address widespread deficiency.

Addressing this modifiable risk factor through coordinated prevention and awareness efforts can significantly contribute to reducing the burden of vitamin D deficiency–related morbidity and improving overall population health.

## Data Availability

The original contributions presented in the study are included in the article/supplementary material, further inquiries can be directed to the corresponding author.
